# The Arginine Deiminase Pathway Impacts Antibiotic Tolerance during Biofilm-Mediated Streptococcus pyogenes Infections

**DOI:** 10.1128/mBio.00919-20

**Published:** 2020-07-07

**Authors:** Jeffrey A. Freiberg, Yoann Le Breton, Janette M. Harro, Devon L. Allison, Kevin S. McIver, Mark E. Shirtliff

**Affiliations:** a Department of Microbial Pathogenesis, School of Dentistry, University of Maryland, Baltimore, Maryland, USA; b Department of Cell Biology & Molecular Genetics and Maryland Pathogen Research Institute, University of Maryland, College Park, Maryland, USA; c Department of Microbiology and Immunology, School of Medicine, University of Maryland, Baltimore, Baltimore, Maryland, USA; KUMC

**Keywords:** *Streptococcus pyogenes*, antibiotic tolerance, arginine deiminase, biofilms, group A *Streptococcus*

## Abstract

Bacterial biofilms are responsible for a variety of serious human infections and are notoriously difficult to treat due to their recalcitrance to antibiotics. Further work is necessary to elicit a full understanding of the mechanism of this antibiotic tolerance. The arginine deiminase (ADI) pathway is responsible for bacterial pH maintenance and is highly expressed during biofilm growth in multiple bacterial species. Using the group A *Streptococcus* (GAS) as a model human pathogen, the ADI pathway was demonstrated to contribute to biofilm growth. The inability of antibiotics to reduce GAS populations when in a biofilm was demonstrated by *in vitro* studies and a novel animal model of nasopharyngeal infection. However, disruption of the ADI pathway returned GAS biofilms to planktonic levels of antibiotic sensitivity, suggesting the ADI pathway is influential in biofilm-related antibiotic treatment failure and provides a new strategic target for the treatment of biofilm infections in GAS and potentially numerous other bacterial species.

## Abstract (precis)

Biofilm-mediated bacterial infections are a major threat to human health because of their recalcitrance to antibiotic treatment. Through the study of Streptococcus pyogenes, a significant human pathogen that is known to form antibiotic-tolerant biofilms, we demonstrated the role that a bacterial pathway known for responding to acid stress plays in biofilm growth and antibiotic tolerance. This not only provides some insight into antibiotic treatment failure in S. pyogenes infections but also, given the widespread nature of this pathway, provides a potentially broad target for antibiofilm therapies. This discovery has the potential to impact the treatment of many different types of recalcitrant biofilm infections.

## INTRODUCTION

Bacterial biofilms represent a major human health problem, as the biofilm mode of growth allows bacteria to persist during an infection despite appropriate antibiotic therapy, where bacteria grown in a biofilm can often withstand 1,000-fold higher concentrations of antibiotics than their planktonic counterparts (1). While multiple factors have been suggested to contribute to this phenomenon, we still lack satisfactory answers to the question of why antibiotics fail to eliminate biofilms (2). Streptococcus pyogenes (group A *Streptococcus*; GAS) is a significant human pathogen responsible for over 500,000 deaths annually (3). Despite being readily susceptible to antibiotics in planktonic form, GAS has been shown to form biofilms, both *in vitro* and *in vivo*, that are associated with antibiotic tolerance and treatment failure (4–6).

Recent work from our laboratory identified an association between GAS biofilm growth and increased expression of the arginine deiminase (ADI) pathway both *in vitro* and *in vivo* (7, 8). The ADI pathway is employed by many bacteria to buffer against acidic environments (9). It is highly expressed in the biofilms of multiple different bacteria (10–14) and has been shown to directly influence biofilm growth by regulating pH in Staphylococcus epidermidis (15).

The *arc* operon, which encodes all the components of the ADI pathway in GAS, functions to raise intracellular pH by releasing two moles of ammonia for every mole of l-arginine degraded (see Fig. S1A in the supplemental material). Like many other lactic acid bacteria, GAS has an intricate *arc* operon (Fig. S1B). While the *arc* operon already has an established role in tolerance to acid stress (16–18), a link between the *arc* operon and antibiotic tolerance has also been suggested. Caldelari et al. (19) reported that penicillin-tolerant Streptococcus gordonii mutants had increased levels of ArcA and ArcB expression, while Chaussee et al. (20) found that mutation of the transcriptional regulator Rgg in S. pyogenes results in a penicillin-tolerant mutant characterized, in part, by increased expression of the *arc* operon. Moreover, upregulation of *arcA* restored antibiotic tolerance to a susceptible *Enterococcus* strain (21).

10.1128/mBio.00919-20.1 FIG S1 Overview and construction of arginine deiminase pathway mutants. (A) The ADI pathway involves three enzymes, arginine deiminase (ArcA), ornithine carbamoyltransferase (ArcB), and carbamate kinase (ArcC), that work in conjunction to convert l-arginine into l-citrulline, l-citrulline into carbamoyl phosphate and l-ornithine, and finally carbamoyl phosphate into CO_2_ and ATP. This process raises the pH by releasing two moles of ammonia for every mole of l-arginine degraded, which, in turn, reduces acid stress. (B) The *arc* operon in wild-type Streptococcus pyogenes M1T1 strain 5448. ArcA, ArcB, and ArcC are encoded by the *arc* operon, which also encodes a putative arginine-ornithine antiporter (ArcD), dipeptidase (ArcT), and acetyltransferase (SP5448_RS03095). Arrows indicate the insertion site for the pSinS-derived plasmid used to make each mutant. 5448-NC is WT-NC, an isogenic 5448 WT strain with spectinomycin resistance as a result of insertion of the pSinS plasmid in a noncoding region outside the *arc* operon. Download FIG S1, TIF file, 1.3 MB. Copyright © 2020 Freiberg et al. 2020 Freiberg et al. This content is distributed under the terms of the Creative Commons Attribution 4.0 International license.

Because the production of Arc proteins is significantly elevated during biofilm growth (7, 8), we hypothesized that the arginine deiminase pathway influences biofilm formation by GAS. Furthermore, because biofilm formation is associated with antibiotic tolerance in GAS (4–6), we also hypothesized that the arginine deiminase pathway contributes to biofilm-associated antibiotic tolerance. To test these hypotheses, we constructed mutants with deficiencies in the arginine deiminase pathway and assayed their growth in the biofilm mode *in vitro* and their antibiotic tolerance *in vivo* using an animal model. Our results demonstrate that the *arc* operon of GAS has a significant effect on biofilm growth and antibiotic tolerance *in vitro* and *in vivo*. This represents the first report of a connection among a genetic component, biofilm growth, and antibiotic treatment failure in the group A *Streptococcus.*

## RESULTS

### The arginine deiminase pathway is transcribed as a single operon.

To assess the role of the arginine deiminase pathway in GAS biofilm growth, we constructed a strain with a mutation in the *arc* operon (see Table S1 and Fig. S1 in the supplemental material). Although the *arc* operon is predicted to be transcribed as two separate operons of three genes each in the M1T1 GAS strain 5448 (8), we found that insertion of a suicide plasmid (pSinS) into the beginning of *arcA* inactivated the expression of *arcA*, *arcB*, *arcD*, and *arcC* (*arc* mutant; JF155) (Fig. S2). Although we did not specifically test expression of *arcT*, we presume that the entire *arc* operon is transcribed as a single operon, since insertion of pSinS into the beginning of *arcA* resulted in inactivation of both the first and last gene in the operon (*arcA* and *arcC*) (Fig. S2).

10.1128/mBio.00919-20.2 FIG S2 Quantitative real-time PCR-derived transcription levels for genes in and adjacent to the *arc* operon. Transcription levels for each gene were normalized to *gyrA* expression levels for the *arc* mutant (JF155) and then expressed as a percentage of the expression level in the WT (5448). Data presented represent the means ± SD from two independent experiments. Download FIG S2, TIF file, 0.6 MB. Copyright © 2020 Freiberg et al. 2020 Freiberg et al. This content is distributed under the terms of the Creative Commons Attribution 4.0 International license.

10.1128/mBio.00919-20.5 TABLE S1 GAS strains and plasmids used in this study. Download Table S1, DOCX file, 0.02 MB. Copyright © 2020 Freiberg et al. 2020 Freiberg et al. This content is distributed under the terms of the Creative Commons Attribution 4.0 International license.

### The arginine deiminase pathway does not have an important role in exponential-phase planktonic growth.

**FIG 1:**
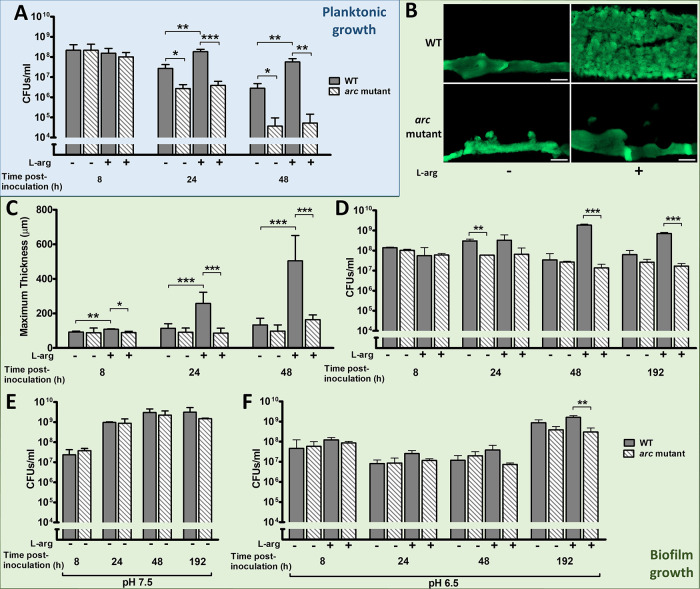
Comparison of WT and Arc mutant GAS during planktonic and biofilm growth. (A) Number of CFU/ml of the WT and *arc* mutant strains at indicated time points after inoculation of a planktonic culture in THY-B both with and without supplementation with 10 mM l-arginine. (B) Representative cross sections of WT and *arc* mutant biofilms formed on unbuffered THY-A after 144 h. The white bar represents 100 μm. (C) Maximum biofilm thickness measured as outlined in Materials and Methods using either COMSTAT2 or ImageJ to analyze images obtained by confocal microscopy at the indicated time points. (D to F) Number of CFU/ml of the WT and *arc^–^* mutant strains at indicate time points after inoculation of a biofilm culture on THY-A plates with and without supplementation with 10 mM l-arginine. (D) Unbuffered THY-A with and without supplementation with 10 mM l-arginine. (E) THY-A buffered at pH 7.5. (F) THY-A buffered at pH 6.5 with and without supplementation with 10 mM l-arginine. Data presented represent the means ± standard deviations (SD). *, *P* < 0.05; **, *P* < 0.01; ***, *P* < 0.001.

To determine the role of the *arc* operon during planktonic growth, the *arc* mutant was grown in Todd Hewitt plus yeast extract (THY) broth both with and without supplementation with 10 mM ADI substrate and l-arginine (THY_L-arg_). The *arc* mutant and the wild-type (WT) strain (5448) displayed similar growth curves and doubling times (Fig. S3), along with similar numbers of CFU/ml at early stationary time points (Fig. 1A). Although there was a decrease in the number of CFU/ml in both strains after 24 h, there was a significantly greater decrease in the *arc* mutant strain at both 24 and 48 h (Fig. 1A). The addition of l-arginine did not make a difference for any time point less than 24 h. However, the presence of excess l-arginine led to greater survival at 24 and 48 h in the WT strain but not the *arc* mutant (Fig. 1A).

10.1128/mBio.00919-20.3 FIG S3 Growth curve analysis of WT and *arc* mutant. Planktonic cultures of the WT and the *arc* mutant were grown in THY-B. Optical density was monitored using a Klett-Summerson photoelectric colorimeter and reported here in arbitrary Klett units. Data presented represent the means ± SEM from three independent experiments. Download FIG S3, TIF file, 0.6 MB. Copyright © 2020 Freiberg et al. 2020 Freiberg et al. This content is distributed under the terms of the Creative Commons Attribution 4.0 International license.

### Regulation of pH by the arginine deiminase pathway enhances biofilm growth in the presence of excess arginine.

Given that the Arc proteins are highly upregulated during GAS biofilm growth (8), we anticipated seeing a very dramatic effect on biofilm production in the *arc* mutant. However, a predominant phenotype was not obvious when we grew GAS biofilms on polycarbonate membrane filters on top of THY agar (Fig. 1B). In fact, there was only a modest decrease in biofilm biomass and thickness in the mutant strain and only a slight reduction in the total number of CFU (Fig. 1C and D). In contrast, a significant difference was seen when the two strains were grown on THY plates supplemented with excess l-arginine (THY_L-arg_). The presence of excess arginine made no difference to the mutant strain, but there were significant increases in the thickness and total number of CFU in the WT strain (Fig. 1B to D).

Since the ADI pathway is known to be responsible for acid stress response in GAS (16–18), we tested whether this enhancement of biofilm growth was related to pH regulation. To study the effect of pH, we used buffered media as opposed to the unbuffered THY media used in earlier experiments (which has a starting pH of 7.8). Biofilm formation and growth of the WT and the *arc* mutant strains were compared on media buffered at pH 6.5 (THY_6.5_), pH 7.5 (THY_7.5_), and pH 6.5 supplemented with 10 mM l-arginine (THY_6.5+L-arg_). pH 6.5 was chosen as it represents both acidic conditions and is also the optimal pH for activity of ArcA and ArcB (22). When grown as biofilms on THY agar at pH 7.5 (Fig. 1E), both strains resembled the WT strain grown in the presence of excess arginine. When the two strains were grown as biofilms on THY agar at pH 6.5 (Fig. 1F), they both resembled the *arc* mutant grown on unbuffered media at early time points. However, as opposed to what was seen in Fig. 1D, both strains showed increased numbers of CFU at 192 h versus 48 h when grown at pH 6.5.

Finally, the addition of excess arginine to buffered medium was tested. When grown on THY_6.5+L-arg_ (Fig. 1F), the WT strain showed significantly enhanced growth versus the *arc* mutant. This suggests that the WT strain’s ability to utilize the excess arginine allows it to better compensate for the effects of media buffered at an acidic pH.

### The *arc* operon contributes to antibiotic tolerance only during biofilm growth.

**FIG 2:**
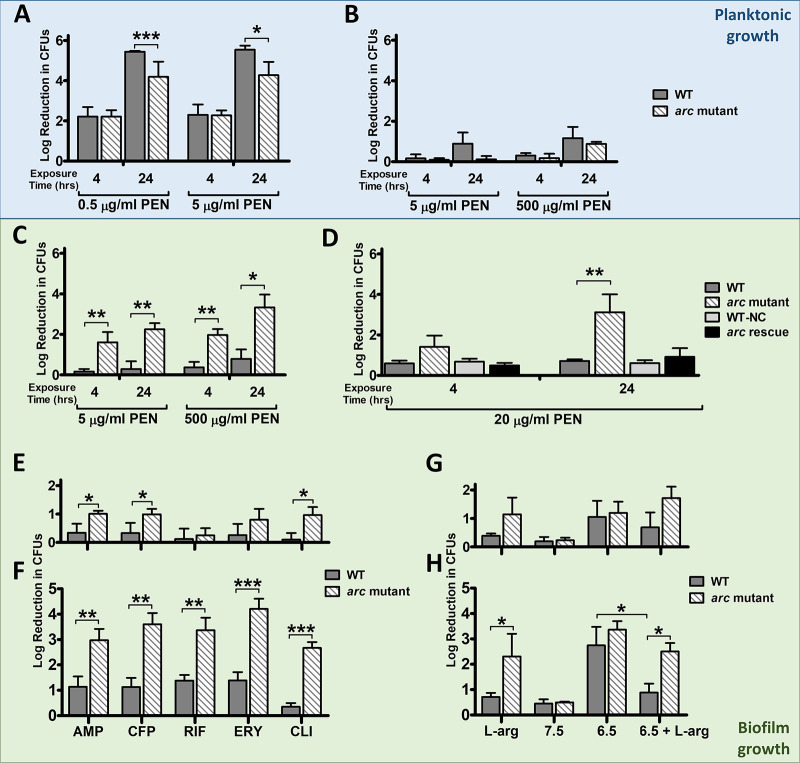
Penicillin tolerance of WT and Arc mutant GAS. Planktonic cultures of the WT strain and *arc* mutant were grown either to mid-log phase (approximately 4 to 5 h) (A) or for 24 h (B) and exposed to the indicated concentrations of penicillin (PEN) for 4 and 24 h. (C to H) Biofilm cultures of the WT strain and *arc* mutant (C and E to H) or the WT strain, *arc* mutant, WT-NC, and *arc* mutant rescue strain (D) were grown for 48 h and exposed to the indicated concentrations of penicillin for 4 and 24 h. (E and F) Biofilm cultures of the WT and *arc* mutant strains were grown for 48 h and exposed to the following concentrations of the indicated antibiotic for either 4 h (E) or 24 h (F): AMP (ampicillin, 5 μg/ml), CFP (cefoperazone, 150 μg/ml), RIF (rifampin, 7 μg/ml), ERY (erythromycin, 2 μg/ml), and CLI (clindamycin, 14 μg/ml). (G and H) Biofilm cultures of the WT and *arc* mutant strains were grown for 48 h on either THY-A_L-arg_, THY-A_7.5_, THY-A_6.5_, or THY-A_6.5+L-arg_ and then exposed to 20 μg/ml penicillin G for either 4 h (G) or 24 h (H). Data presented represent the means ± SD from three independent experiments measuring either the log_10_ fold reduction in CFU versus an untreated culture (log fold reduction_untreated_ or log fold reduction_biofilm_). *, *P* < 0.05; **, *P* < 0.01; ***, *P* < 0.001.

Since increased tolerance to antibiotics is a key characteristic of biofilms (2) and the *arc* operon had previously been implicated in penicillin tolerance in S. gordonii (19), we tested the antibiotic tolerance of the *arc* mutant strain. During planktonic growth, there was no increase in penicillin susceptibility in the *arc* mutant compared to the WT strain (Fig. 2A and B), even when excess arginine was added (Fig. S4). Mid-log-phase bacteria were still predictably highly susceptible to penicillin at both 0.5 and 5 μg/ml. As expected, overnight stationary-phase bacteria were much less susceptible to penicillin, because penicillin does not kill GAS in stationary phase (23). However, when grown as biofilms, the two strains showed significant differences in their penicillin tolerance (Fig. 2C). The WT strain had decreased susceptibility to penicillin, even after 24 h of penicillin exposure, while the *arc* mutant displayed penicillin susceptibility that more closely resembled that of planktonic GAS. This effect was seen both at a 5 μg/ml concentration of penicillin and also at 500 μg/ml. Given that there was no significant difference between 5 and 500 μg/ml penicillin, we used 20 μg/ml for all subsequent experiments, as it represents the peak serum concentration during antibiotic therapy (24).

10.1128/mBio.00919-20.4 FIG S4 Penicillin tolerance of WT and *arc* mutant during planktonic growth in excess l-arginine. Planktonic cultures of the WT and *arc* mutant strains were grown in THY_L-arg_ to mid-log phase (approximately 4 to 5 h). Data presented represent the means ± SD from three independent experiments measuring the log_10_ fold reduction in CFUs versus an untreated culture (log fold reduction_untreated_). *, *P* < 0.05; **, *P* < 0.01. Download FIG S4, TIF file, 0.6 MB. Copyright © 2020 Freiberg et al. 2020 Freiberg et al. This content is distributed under the terms of the Creative Commons Attribution 4.0 International license.

The mutant’s penicillin susceptibility was confirmed to be due to disruption of the *arc* operon, as insertion of the pSinS plasmid in a noncoding region outside the *arc* operon (WT-NC, 5448-NC; Fig. S1) did not have any effect, and rescue of the *arc* mutation (*arc* rescue strain; JF155-RS) reversed the observed phenotype (Fig. 2D). Importantly, disruption of the *arc* operon also made GAS more susceptible to multiple different antibiotics. The *arc* mutant strain showed decreased biofilm survival in the presence of ampicillin, cefoperazone, rifampin, erythromycin, and clindamycin, while the WT biofilm was tolerant to all antibiotics tested (Fig. 2E and F).

### Antibiotic tolerance mediated by the arginine deiminase pathway is dependent on pH regulation.

As was the case of enhancement in biofilm growth, the increased antibiotic tolerance during biofilm growth was linked to the *arc* operon’s ability to respond to acid stress. When the WT and the *arc* mutant strains were grown in THY_7.5_, there was no difference in terms of penicillin sensitivity; both strains formed biofilms that were highly resistant to killing by penicillin (Fig. 2G and H). However, when the two strains were grown at pH 6.5, they were both highly susceptible to penicillin. While the addition of excess arginine allowed the WT strain to overcome the effects an acidic pH, the *arc* mutant remained highly susceptible to penicillin because it was unable to utilize arginine (Fig. 2G and H).

### Loss of the arginine deiminase pathway reduces *in vivo* biofilm formation in a murine model of NALT infection.

**FIG 3:**
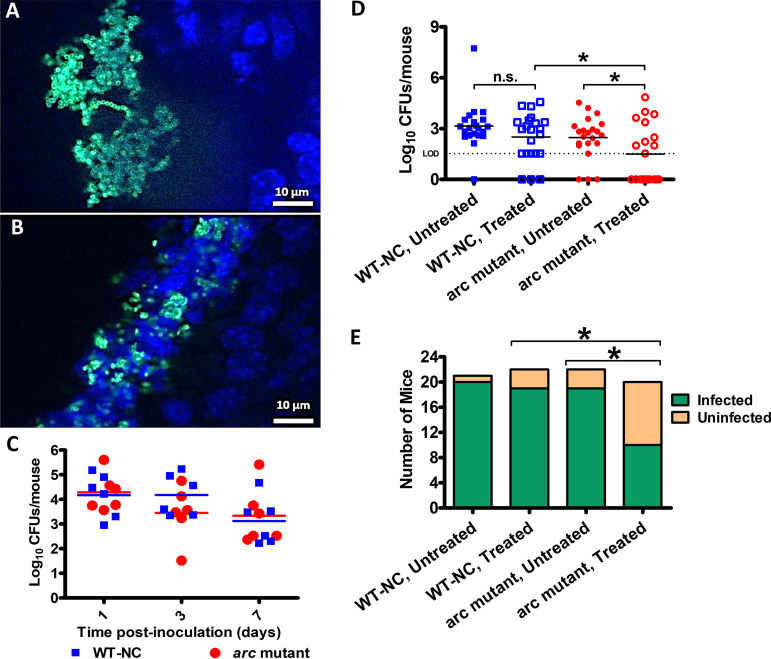
Colonization of C57BL/6 IL-17A^−/−^ mouse nasal tissues in the NALT infection model and penicillin tolerance. Nasal tissues harvested from mice at 1 day postinoculation (dpi) were cryosectioned and stained with FITC-conjugated anti-GAS antibody (green) and DAPI (blue) to visualize the formation of GAS microcolonies within the NALT of C57BL/6 IL-17A^−/−^ mice inoculated with either WT-NC (A) or the *arc* mutant (B). (C) Nasal tissues were harvested at the indicated time points and cultured to determine the total number of CFU/mouse present. *n *= 6 mice per bacterial strain per time point. Mice inoculated intranasally with WT-NC or the *arc* mutant were treated with penicillin G or a placebo. (D) Nasal tissues were harvested 2 days after treatment (5 dpi) and cultured to determine the total number of CFU/mouse present. (E) Mice were determined to have cleared the infection if their CFU count was below the limit of detection (33 CFU/mouse). *n *= 20 to 22 mice per group. Data represent the combination of 5 independent experiments. Statistical significance was determined by a two-tailed Fisher’s exact test. *, *P* < 0.05; n.s., not significant.

Since the Arc proteins were previously shown to be expressed and highly immunogenic in an *in vivo* model of a biofilm-mediated GAS infection (7), we wanted to test the importance of the *arc* operon during *in vivo* biofilm formation using a murine GAS nasal infection model. In mice, GAS is known to colonize the nasal-associated lymphoid tissue (NALT), which can be considered analogous to the colonization of human tonsils (25). Because mice deficient in IL-17A have been shown to have higher rates of GAS colonization for longer durations (26, 27), we used IL-17A knockout mice (IL-17A^−/−^) (28). Mice were inoculated intranasally with a total of 1 × 10^8^ CFU of either WT-NC (5448-NC), an isogenic 5448 WT strain with spectinomycin resistance, or the *arc* mutant strain. Cryofixation of the infected nasal tissue and staining with a GAS-specific fluorescein isothiocyanate (FITC)-tagged antibody revealed the formation of microcolonies in the NALT of mice inoculated with the wild-type strain WT-NC as early as 24 h postinoculation (Fig. 3A). Mice inoculated with the *arc* mutant, however, lacked the dense microcolonies seen in the WT strain and instead exhibited smaller clusters of more isolated bacteria (Fig. 3B). Despite this difference in biofilm formation, both strains displayed similar numbers of CFU (Fig. 3C), suggesting the difference in phenotype was due to a defect in biofilm formation and not an overall growth deficiency.

### The *arc* operon is important for biofilm penicillin tolerance *in vivo*.

To test whether the ADI pathway was involved in penicillin tolerance *in vivo*, we adapted the NALT infection model to include a penicillin treatment step. Three days (72 h) after initial intranasal inoculation with GAS, mice received either a single intramuscular dose of penicillin G or sterile saline as a control. Two days (48 h) following the penicillin treatment, nasal tissue was collected and plated to determine the number of CFU remaining (Fig. 3D). In the absence of penicillin treatment, no difference in clearance was observed between mice infected with the WT-NC strain and those infected with the *arc* mutant. When the mice infected with WT GAS were treated with penicillin, there was no improvement in clearance rates versus the untreated controls. However, the mice infected with the *arc* mutant strain showed a significant increase in clearance after penicillin treatment (Fig. 3E), suggesting that the *arc* operon is important for penicillin tolerance during an *in vivo* biofilm infection.

## DISCUSSION

The arginine deiminase pathway is an important mechanism for adaptation to acidic environments in a number of bacterial taxa, including GAS (16, 29). While this pathway is upregulated in multiple models of biofilm growth (7, 8, 10–14), this is the first demonstration of a definitive role for the ADI pathway during GAS biofilm growth. Furthermore, this is, to our knowledge, the first evidence of a genetic component to the increased antibiotic tolerance observed in GAS biofilms.

Using targeted mutations, we demonstrated the importance of the ADI pathway during GAS biofilm growth. Similar to previous studies in planktonic cells (16–18), a fully intact ADI pathway was required for GAS to utilize arginine for protection against acid stress. An *arc* mutant showed significantly reduced biofilm formation in the presence of excess arginine compared with the WT strain and was unable to form a robust biofilm in an acidic environment, even in the presence of excess arginine (Fig. 1). This is notable, since GAS biofilm growth is associated with rapid acidification to a pH lower than 6.0 *in vitro* (30). Interestingly, as opposed to Fig. 1D, where unbuffered THY medium was used, both strains showed increased growth at 192 h versus 48 h when the medium was buffered at pH 6.5 (Fig. 1F). It is unclear whether this is the result of selection pressure for more acid-tolerant variants over the duration of the experiment or due to some other unrecognized mechanisms for dealing with mild acid stress in GAS.

Although *arc* operon mutations reportedly exhibit considerable effects on energy metabolism and resisting acid stress during planktonic growth, we did not see comparable effects during the exponential or early stationary phase. These discrepancies were likely due in part to differences in the growth conditions among the studies. Degnan et al. (16) found decreased survival of *arcA* mutants within 6 h but only in media artificially acidified to pH 5 or lower. Cusumano et al. (17) reported decreased final growth yields in *arc* operon mutants, but, similar to our findings, there was no difference in the growth curve or doubling time between the mutants and the WT strain. In addition, Cusumano et al. (17) used low-glucose C medium, as opposed to the glucose-rich THY medium employed in our study. Glucose represses expression of the *arc* operon in GAS (31–33), which may explain the lack of an effect in the *arc* mutant until late stationary phase (Fig. 1A). The reduced survival of the *arc* mutant at 24 and 48 h fits in the context of delayed glucose depletion in THY medium leading to derepression of the *arc* operon at a later time point than would be seen in C medium. However, our earlier work demonstrated that although transcription of the *arc* operon is increased by late log/early-stationary-phase time points, the level of production of Arc proteins (ArcA, ArcB, and ArcC) even at 48 h into stationary phase is significantly lower than that during biofilm growth (8).

Although the arginine deiminase pathway is involved in the response to acid stress during stationary-phase growth, its role in antibiotic tolerance is specific to biofilm growth. Not only did the arginine deiminase pathway fail to influence penicillin susceptibility during exponential growth (Fig. 2A), but stationary-phase bacteria demonstrated marked resistance to killing by penicillin irrespective of the functional status of the *arc* operon (Fig. 2B). This characteristic antimicrobial tolerance of stationary-phase bacteria has been thought to contribute, to some degree, to the resistance of biofilms (34). However, in contrast to stationary-phase cells, the loss of the *arc* operon caused a sharp increase in the penicillin susceptibility of biofilm cultures (Fig. 2C and D). Although this effect was mediated by the ability of the arginine deiminase pathway to regulate pH (Fig. 2G and H), arginine supplementation did not affect antibiotic tolerance during planktonic growth (Fig. S4).

The role of the *arc* operon in antibiotic tolerance is not limited to penicillin. Two other beta-lactams (ampicillin and cefoperazone), as well as antibiotics of three other classes, all showed a similar effect (Fig. 2E and F). This is particularly surprising as clindamycin and erythromycin are considered to be bacteriostatic. The increased cell death observed with multiple bacteriostatic and bactericidal antibiotics that function at the cell wall and intracellularly suggests that a nontraditional mechanism of antibiotic-mediated killing is functioning in the *arc* mutant biofilms.

We next determined whether the phenotype of GAS biofilm cells observed *in vitro* was replicated *in vivo*. We utilized a murine model of GAS NALT infection because it effectively recapitulates pharyngitis and GAS colonization of human tonsils (25). GAS tonsillopharyngitis is frequently associated with antibiotic treatment failure (35), and evidence of GAS biofilms has been found both in human tonsils (36) and mouse NALT (37). Indeed, we found evidence of microcolony formation in the nasal cavities of the mice (Fig. 3A), persistence of bacteria (Fig. 3B), and phenotypic antibiotic tolerance (Fig. 3C and D), all defining characteristics of a biofilm infection (38). However, the *arc* mutant exhibited reduced microcolony formation compared to that of the WT strain (Fig. 3B), suggesting a deficiency in biofilm formation *in vivo*. Additionally, the increased penicillin susceptibility of *arc* biofilms *in vitro* was recapitulated *in vivo*; the rate of clearance of the *arc* mutant was increased by penicillin treatment (Fig. 3E).

Taken together, our results imply that a unique relationship exists between the role of the ADI pathway in bacterial biofilm growth and antibiotic tolerance. The proteins in the ADI pathway are known to be highly expressed during biofilm growth in GAS and other bacterial species (7, 8, 10–14). While this increase in expression is ostensibly due to a decrease in pH within the biofilm environment, it has the important consequence of increasing tolerance to antibiotics within the biofilm. Although this increased antibiotic tolerance is pH dependent, it is distinct from the role the ADI pathway plays in regulating pH in stationary-phase cultures, given the specificity of the *arc*-dependent tolerance to biofilm cultures. The fact that a pathway as ubiquitous as the ADI pathway has such a profound impact on susceptibility to a number of clinically relevant antibiotics suggests this finding is broadly applicable to multiple bacterial pathogens of humans. We are optimistic that understanding the role the ADI pathway plays in antibiotic tolerance will lead to novel antibacterial therapies. Furthermore, unlike most of the current therapies for treating biofilm infections that require dispersing or mechanically breaking up biofilms before eradicating bacteria (38–40), disrupting the ADI pathway has the potential to finally provide a mechanism for the direct targeting of bacteria while they are still within a biofilm.

## MATERIALS AND METHODS

### Bacterial strains and growth conditions.

The previously described M1T1 GAS strain 5448 was used in this study (41). GAS was routinely grown at 37°C in Todd Hewitt broth (BD Laboratories) supplemented with 0.2% yeast extract (Sigma) (THY-B), except when otherwise noted. Solid culture medium (THY-A) was obtained by adding agar (Fisher Scientific) to THY-B at a concentration of 15 g/liter. Escherichia coli strain TOP10 was used as a host to construct the plasmid vectors required to generate GAS mutants in this study. E. coli was grown in LB broth (Fisher Scientific). When appropriate, antibiotics were used for selection: kanamycin (Sigma) at 50 μg/ml for E. coli and 300 μg/ml for S. pyogenes; spectinomycin (Research Products International Corp.) at 100 μg/ml for both E. coli and S. pyogenes. THY_L-arg_ was made by adding a 1 M stock solution of filter-sterilized l-arginine (Sigma) to THY-B to a final concentration of 10 mM. THY_6.5_ was made by making up THY-B in 90% double-distilled water (ddH_2_O) and 10% 1 M stock solution of morpholineethanesulfonic acid (MES; Sigma) buffered to pH 6.5. THY_7.5_ was made by making up THY-B in 90% ddH_2_O and 10% 1 M stock solution of HEPES (Sigma) buffered to pH 7.5. THY_6.5+L-arg_ was made by adding a 1 M stock solution of filter-sterilized l-arginine to THY_6.5_ to a final concentration of 10 mM.

### Construction and rescue of *arc* mutants.

Mutations in the *arc* operon were constructed using the previously described pSin/pHlp mutagenesis system for stable plasmid integration in the GAS chromosome in addition to standard laboratory techniques for genetic manipulation of GAS (42, 43). Briefly, a region, 170 to 360 bp long, downstream of the promoter of the gene of interest was amplified from the 5448 genome using the primers shown in Table S2 in the supplemental material. The resulting PCR fragment was inserted into pSinS, which is a spectinomycin-resistant (Sp^r^) suicide plasmid that only replicates in GAS when the pHlpK kanamycin-resistant (Km^r^) temperature-sensitive helper plasmid is present. The resulting construct was transformed into pHlpK-containing 5448, and Sp^r^ Km^r^ transformants were selected at 30°C, the permissive temperature for replication of the pHlpK plasmid. Transformants were then grown at the nonpermissive temperature of 37°C in the presence of spectinomycin to select for clones with chromosomal integration of the mutagenic plasmid. Loss of the pHlpK plasmid was confirmed (Sp^r^ Km^s^) and chromosomal integration at the site of homology was confirmed by PCR.

10.1128/mBio.00919-20.6 TABLE S2 Primers used in this study. Download Table S2, DOCX file, 0.01 MB. Copyright © 2020 Freiberg et al. 2020 Freiberg et al. This content is distributed under the terms of the Creative Commons Attribution 4.0 International license.

To rescue the *arc* mutation, the pHlpK plasmid was reintroduced into the mutant strain with kanamycin and spectinomycin selection at 30°C. Transformants were subsequently moved to 37°C, and Sp^s^ Km^s^ clones representing excised suicide plasmid were identified by replica plating and confirmed by PCR. The strains constructed for this study are detailed in Table S1. In addition, a noncoding region outside the *arc* operon was targeted for pSinS-mediated integration to make the control strain 5448-NC, which is an Sp^r^ variant of 5448 where the pSinS integration has no effect on *arc* operon transcription.

### qRT-PCR.

RNA was isolated from early-stationary-phase GAS cultures using the Isolate II RNA minikit (Bioline), and contaminating DNA was removed using the TURBO DNA-free kit (Ambion) according to the manufacturer’s directions. Transcript levels were quantified using the quantitative reverse transcription-PCR (qRT-PCR) probes listed in Table S2 and the iTaq universal SYBR green one-step kit (Bio-Rad) according to the manufacturer’s instructions. The qRT-PCRs were performed using an iQ5 real-time PCR detection system (Bio-Rad). Expression values were calculated by the ΔΔ*C_T_* method, with *gyrA* mRNA levels used for normalization.

### Planktonic growth conditions.

Overnight planktonic cultures of GAS were diluted 1:100 into prewarmed THY-B in side-arm flasks to allow for the monitoring of optical density without adding additional oxygen to the culture. Cell growth was monitored using a Klett-Summerson photoelectric colorimeter. At designated time points, sample aliquots were removed from the culture flask to enumerate the number of viable bacteria via serial dilution. Ten microliters of 10-fold serial dilutions of the samples were plated in triplicate on THY-A and incubated for 24 h at 37°C to determine the number of CFU per ml.

### Biofilm growth conditions.

Stationary biofilms were grown on membrane filters in a manner similar to that of Anderl et al. (44). Briefly, sterile, polycarbonate membrane filters (19-mm diameter; 0.2-μm pore size) (Whatman) were placed on top of THY-A agar culture medium. Overnight planktonic cultures of GAS were diluted 1:100 in THY-B, and 10 μl of the dilution was used to inoculate the center of a filter. The agar plates containing the filters were incubated at 37°C, 5% CO_2_. Filters were transferred to fresh THY-A plates every 24 h. At designated time points, filters were removed from the agar and either prepared for confocal microscopy or used to determine the number of viable bacteria. Filters used to enumerate the number of viable bacteria were placed into 8 ml of sterile phosphate-buffered saline (PBS) (Sigma) and homogenized on ice for 40 s to disrupt the biofilms prior to plating serial dilutions. Ten microliters of 10-fold serial dilutions of the homogenized samples were plated in triplicate on THY-A and incubated for 24 h at 37°C to determine the number of CFU per filter. When indicated, THY-A plates were replaced with THY-A_L-arg_, THY-A_6.5_, THY-A_7.5_, or THY-A_6.5+L-arg_ plates.

### *In vitro* biofilm imaging.

Filters containing biofilms to be used for confocal microscopy were transferred to glass slides and incubated for 15 min in the dark with the SYTO 9 stain provided in the Live/Dead *Bac*Light bacterial viability kit (Molecular Probes) according to the manufacturer’s recommended concentrations. After 15 min, the stain was removed with a pipette, VECTASHIELD mounting medium (Vector Laboratories) was added, and the samples were coverslipped. The slides were imaged with a Zeiss LSM 510 Meta confocal scanning laser microscope (Carl Zeiss). A total of 3 z-stacks were obtained at random from 2 biological replicates for each sample. These z-stacks were analyzed to determine biofilm biomass and average thickness using the program Comstat2 (www.comstat.dk) (45, 46). Biofilms older than 16 h were too thick to be imaged by a top-down confocal approach. Instead, older biofilms were embedded in Tissue-Tek optimum cutting temperature (O.C.T.) compound (Sakura Finetek USA), frozen on dry ice, and then stored at –80°C prior to sectioning. O.C.T. embedded biofilms were cross-sectioned using a Leica CM1950 cryostat (Leica Biosystems) and mounted onto Tissue Path Superfrost Plus gold slides (Thermo Scientific) and stored at –20°C prior to staining with SYTO 9 as outlined above. A total of 3 cross sections from 2 biological replicates for each sample were measured to determine the maximum thickness of the biofilm using ImageJ (47).

### Planktonic penicillin susceptibility.

Planktonic cultures were grown as described above. For each culture, once the culture reached mid-log phase (50 to 60 Klett units), three 40-ml aliquots were removed and penicillin G (Sigma) was added. Penicillin G (dissolved in ddH_2_O) was added to two of the aliquots at a concentration of either 0.5 μg/ml or 5 μg/ml, while the third aliquot received no penicillin as a control (an equivalent volume of ddH_2_O was added as a control). An additional sample was taken and serial dilutions were plated to determine the starting number of CFU prior to administration of penicillin. At 4 and 24 h after the addition of penicillin, samples were taken to enumerate the number of viable bacteria remaining. Prior to plating serial dilutions, the samples were pelleted by centrifugation to remove the penicillin and then resuspended in an equal volume of PBS. Serial dilutions were determined as described above for planktonic cultures. Susceptibility to penicillin in planktonic cultures is reported as a log fold reduction either versus the initial concentration of cells or versus the untreated control. These numbers are represented by the following formulas:

$${\text{log fold reduction}}_{\text{original}}=-{\text{log}}_{\text{10}}\frac{\text{CFU/ml in treated sample}}{\text{CFU/ml prior to addition of penicillin}}$$

$${\text{log fold reduction}}_{\text{untreated}}=-{\text{log}}_{\text{10}}\frac{\text{CFU/ml in treated sample}}{\text{CFU/ml in untreated sample}}$$

The same method described above was also repeated with aliquots taken from a late stationary-phase culture (24 h after inoculation), but penicillin concentrations of 5 μg/ml and 500 μg/ml were used instead.

### Biofilm antibiotic susceptibility.

Filter biofilms were grown as described above. After 48 h of growth, the membrane filters containing the biofilms were transferred to either fresh THY-A plates or fresh THY-A plates containing antibiotics (Sigma) at one of the following concentrations: penicillin G (5 μg/ml, 20 μg/ml, or 500 μg/ml), ampicillin (5 μg/ml), cefoperazone (150 μg/ml), rifampin (7 μg/ml), erythromycin (2 μg/ml), and clindamycin (14 μg/ml). After 4 or 24 h of antibiotic exposure, the filters were removed to enumerate the number of viable bacteria as described above for membrane filters. Susceptibility to penicillin in biofilm cultures is reported as log fold reduction versus the untreated control, as represented by the following formula:

$${\text{log fold reduction}}_{\text{biofilm}}=-{\text{log}}_{\text{10}}\frac{\text{CFU/filter in treated sample}}{\text{CFU/filter in untreated sample}}$$

When indicated, THY-A plates were replaced with THY-A_L-arg_, THY-A_6.5_, THY-A_7.5_, or THY-A_6.5+L-arg_ plates with and without antibiotics.

### Animals.

A mix of male and female mice aged 6 to 8 weeks were used in this study, with each group containing equal numbers of each gender. C57BL/6J IL-17A^−/−^ mice were obtained from Yoichiro Iwakura (University of Tokyo, Japan) (28). All mice were housed in the University of Maryland, Baltimore, Dental School Animal Biosafety Level 2 facility. Animal experiments were approved by the University of Maryland, Baltimore, Institutional Animal Care and Use Committee.

### Murine NALT infection model.

Mice were anesthetized with isoflurane, followed by an intraperitoneal injection of 0.1 ml of a ketamine (20 mg/ml) and xylazine (2 mg/ml) solution. Mice were inoculated in each nostril with 2.5 μl of a 2 × 10^10^ CFU/ml solution of GAS for a total of 1 × 10^8^ CFU/mouse. At designated time points, mice were euthanized to harvest their nasal tissue. Euthanized mice were decapitated and nasal tissue was extracted by following the previously described method (48), with the exception that the NALT was kept with the rest of the nasal tissues (nasal turbinates, septum, lateral walls, and palate) for further processing. The dissected nasal tissue was placed in 1 ml PBS and homogenized on ice for 90 s prior to plating 10-fold serial dilutions. Dilutions were plated in triplicate on both THY-A plus spectinomycin (100 μg/ml) and Trypticase soy agar with 5% sheep blood (BD Laboratories). Total numbers of CFU/mouse were determined based on CFU counts on the THY-A plus spectinomycin plates. The identity of colonies as S. pyogenes was confirmed by the presence of betahemolysis on blood agar and by latex agglutination using the Streptocard acid latex test kit (BD Laboratories).

### Penicillin treatment of murine NALT infection model.

Three days postinoculation (dpi) with GAS using the above-described NALT infection model, mice were weighted and given a single 50,000-U/kg of body weight dose of penicillin G (Henry Schein Animal Health) in the form of a 50-μl intramuscular injection into their left thigh. Mice exhibiting signs of systemic disease (weight loss greater than 20%) prior to antibiotic treatment were euthanized in accordance with the University of Maryland, Baltimore, Institutional Animal Care and Use Committee’s guidelines on humane endpoints in rodents. This resulted in fewer than 22 mice for two of the groups. After an additional 48 h (5 dpi), all mice were euthanized to harvest their nasal tissue for CFU counts as outlined above.

### Imaging of infected nasal tissue.

To image *in vivo* biofilm formation, nasal tissue was dissected as described above for mice infected with the NALT infection model. The dissected tissue was embedded in Tissue-Tek O.C.T. compound (Sakura Finetek USA), snap-frozen in liquid nitrogen, and then stored at –80°C prior to sectioning. O.C.T. embedded tissue samples were sectioned using a Leica CM1950 cryostat (Leica Biosystems). Ten-micrometer-thick coronal sections were mounted onto Tissue Path Superfrost Plus gold slides (Thermo Scientific) and stored at –20°C prior to staining. To prepare the samples for staining, the slides were fixed for 5 min with 10% formalin (Sigma), blocked for 30 min at room temperature with 1% bovine serum albumin (Sigma) in PBS (1% BSA–PBS), and rinsed with 0.1% Triton-X (EMD Millipore) in PBS. The slides were then stained for 30 min at 37°C with anti-GAS-FITC-conjugated rabbit antibody (Thermo Scientific) diluted 1:500 in 1% BSA–PBS. The stained slides were rinsed again with 0.1% Triton-X in PBS, counterstained for 30 min at room temperature with 1 μg/ml 4′,6-diamidino-2-phenylindole (DAPI; Sigma), rinsed with ddH_2_O, covered with VECTASHIELD mounting medium, and coverslipped. The slides were then imaged using a Zeiss Axio Imager Z.1 fluorescence microscope with the ApoTome.2 module (Carl Zeiss).

### Statistical analysis.

Data were analyzed using either a two-tailed Student's *t* test or a two-tailed Fisher’s exact test, when appropriate. All statistical analysis was done using GraphPad Prism v5.0 (GraphPad Software). A *P* value of <0.05 was considered significant.
