# Supplementary figures and images for: The Arginine Deiminase Pathway Impacts Antibiotic Tolerance during Biofilm-Mediated Streptococcus pyogenes Infections
Source: mBio. 2020 Jul 7;11(4):e00919-20. doi: 10.1128/mBio.00919-20 (PMC7343988; doi:10.1128/mBio.00919-20)

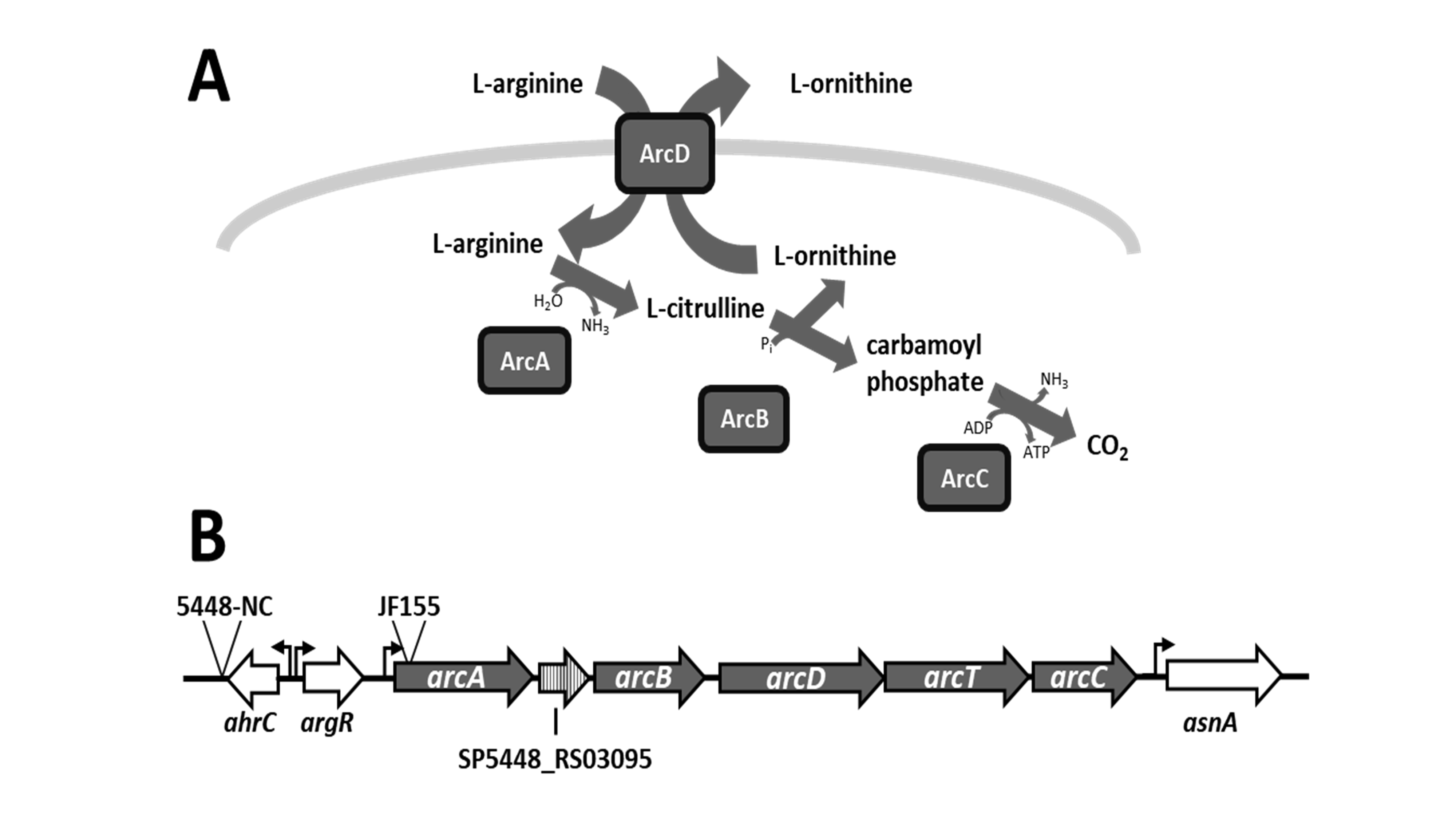

Supplement: FIG S1 [file mBio.00919-20-sf001.tif]

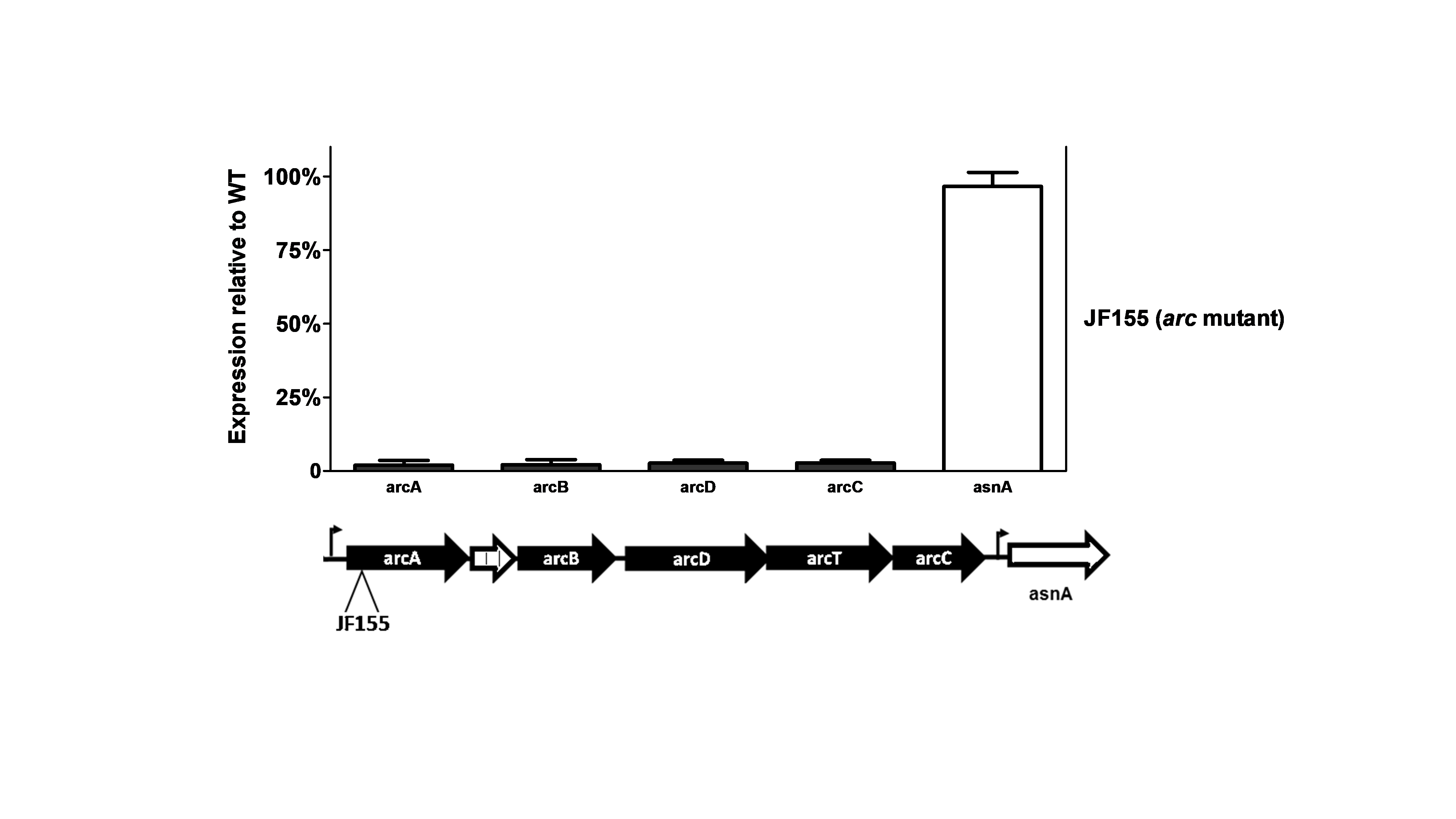

Supplement: FIG S2 [file mBio.00919-20-sf002.tif]

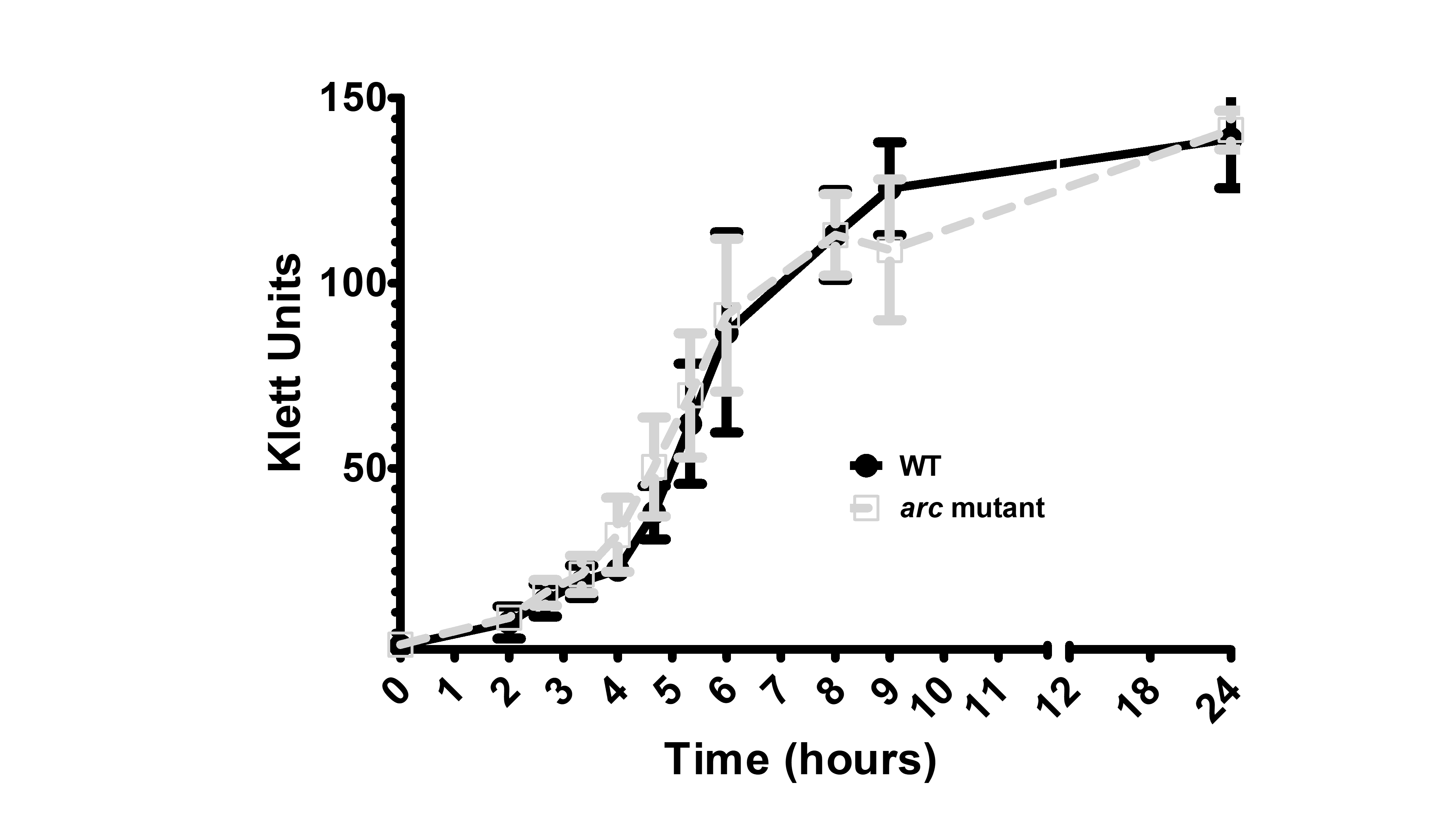

Supplement: FIG S3 [file mBio.00919-20-sf003.tif]

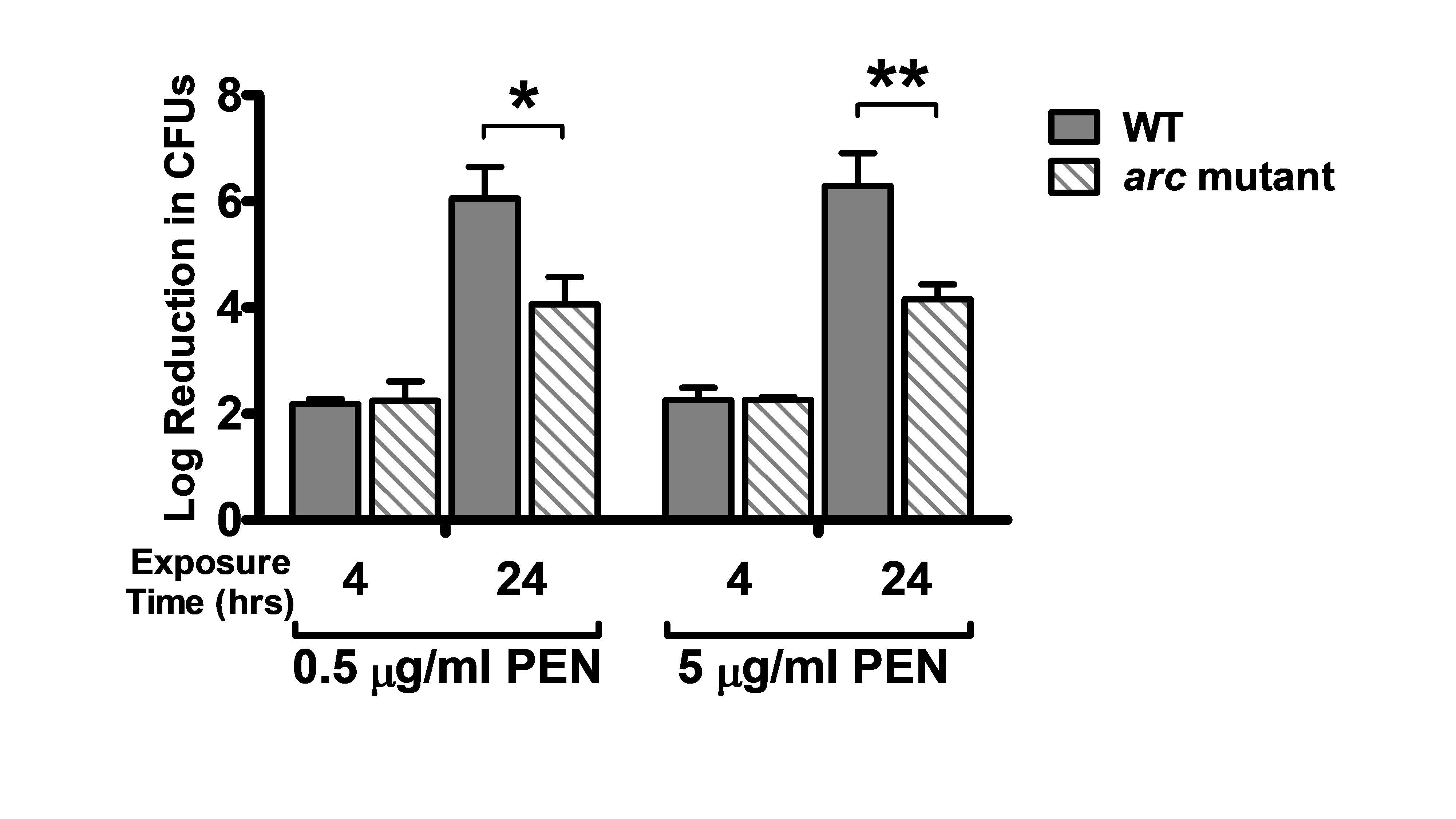

Supplement: FIG S4 [file mBio.00919-20-sf004.tif]
