# Supplementary material for: The Arginine Deiminase Pathway Impacts Antibiotic Tolerance during Biofilm-Mediated Streptococcus pyogenes Infections
Source: mBio. 2020 Jul 7;11(4):e00919-20. doi: 10.1128/mBio.00919-20 (PMC7343988; doi:10.1128/mBio.00919-20)
Supplement: TABLE S1 [file mBio.00919-20-st001.docx]

| **Table S1. GAS strains and plasmids used in this study** | | | | | | |
| --- | --- | --- | --- | --- | --- | --- |
| **Strain name** | | **Phenotype** | | | **Description** | **Reference** |
| 5448 | | wild-type | | | *S. pyogenes* M1T1 strain isolated from invasive disease | (41) |
| JF155 | | 5448-Arc^-^, Sp^R^ | | | 5448-*arcA_188_*::pJAF118 | This study |
| 5448-NC | | 5448, Sp^R^ | | | 5448::pJAF123 (in non-coding region 124 bp after *ahrC.2* stop codon) | This study |
| JF155-RS | | wild-type | | | JF155 with pJAF118 excised and removed | This study |
|  | |  | | |  |  |
| **Plasmid name** | | |  | **Description** | | **Reference** |
|  | pSinS | | | Suicide plasmid for stable insertional inactivation, Sp^R^ | | (42) |
|  | pHlpK | | | Temperature-sensitive conditional helper vector, Km^R^ | | (42) |
|  | pJAF118 | | | Suicide plasmid for mutagenesis of *arcA* | | This study |
|  | pJAF123 | | | Suicide plasmid for mutagenesis of non-coding region downstream of *ahrC.2* | | This study |
|  |  | | |  | |  |
