# Supplementary material for: The Arginine Deiminase Pathway Impacts Antibiotic Tolerance during Biofilm-Mediated Streptococcus pyogenes Infections
Source: mBio. 2020 Jul 7;11(4):e00919-20. doi: 10.1128/mBio.00919-20 (PMC7343988; doi:10.1128/mBio.00919-20)
Supplement: TABLE S2 [file mBio.00919-20-st002.docx]

| **Table S2. Primers used in this study** | |  |
| --- | --- | --- |
| **Primer** | **Description** | **Sequence** |
| 5KOarcA-EcoRI | cloning *arcA_7-187_* into pSinS to make pJAF118-forward primer | GAGGATGAATTCGCTCAAACACCAATTCATGTTTATTC |
| 3KOarcA-HindIII | cloning *arcA_7-187_* into pSinS to make pJAF118-reverse primer | CACGCAAAGCTTGGGCAAAAG |
| 5Up-arcA | to check chromosomal insertion of pJAF118, primer in GAS chromosome | ACTAGGAATATAAGCAAGAGTTAAGAATCTTG |
| 3Down-pSinS | to check chromosomal insertion of pJAF118, primer in pSinS | GAGCGGATAACAATTTCACACAGG |
| 5KOarcD-EcoRI | cloning *arcD_22-378_* into pSinS to make pJAF117-forward primer | GGTTTTAGAATTCCTTCTTCTTACAC |
| 3KOarcD-HindIII | cloning *arcD_22-378_* into pSinS to make pJAF117-reverse primer | GTGAACTAAGCTTGATTAAAATAGCAATCAGCATTTTTTC |
| 5Up-arcD | to check chromosomal insertion of pJAF117, primer in GAS chromosome | GTAAGAGGTAATCATTATGACAGAAG |
| 5KOcontrol-EcoRI | cloning *ahrC.2_298_* to 123 bp after stop codon into pSinS to make pJAF123-forward primer | GAGGTAGAATTCCTGGACTAGCCCAATCCTTTG |
| 3KOcontrol-HindIII | cloning *ahrC.2_298_* to 123 bp after stop codon into pSinS to make pJAF123-reverse primer | GTCTAAGAAGCTTGCGAGTAAACGAATGTCCTGTC |
| 5Up-control | to check chromosomal insertion of pJAF123, primer in GAS chromosome | GCGTTAGCCATTTCACAAACC |
|  |  |  |
| qrtArcAF | qRT-PCR primer for *arcA*, forward primer | CATTTGCCATCGACCCAATGCC |
| qrtArcAR | qRT-PCR primer for *arcA*, reverse primer | CCTTCAATGCGAGTGGTTTCATTACGG |
| qrtArcBF | qRT-PCR primer for *arcB*, forward primer | CATCAACTCGTACGCGTGCAG |
| qrtArcBR | qRT-PCR primer for *arcB*, reverse primer | GCACACCTGAGAATTCTGCCAATTC |
| qrtArcDF | qRT-PCR primer for *arcD*, forward primer | CTCAAATTGGGTGTCTAGCCTCCAC |
| qrtArcDR | qRT-PCR primer for *arcD*, reverse primer | CCTTGTCTGCAACCAATGATTTACTTGGG |
| qrtArcCF | qRT-PCR primer for *arcC*, forward primer | GCCATGACAGAAGGTAGCATCGG |
| qrtArcDR | qRT-PCR primer for *arcC*, reverse primer | GCACCAGACTCCGCCATTTG |
| qrtAsnAF | qRT-PCR primer for *asnA*, forward primer | GCC GCG CTCCTGACTATG |
| qrtAsnAR | qRT-PCR primer for *asnA*, reverse primer | GGGCCTCTTCATCCACACG |
| qrtGyrAF | qRT-PCR primer for *gyrA*, forward primer | CAGTAGTATTACCAGCTCGCTTTCC |
| qrtGyrAR | qRT-PCR primer for *gyrA*, reverse primer | CAGTCGGAAAGTCAGGTCCAG |
